# The role of amphipathic and cationic helical peptides in Parkinson's disease

**DOI:** 10.1002/pro.70020

**Published:** 2024-12-25

**Authors:** Carlos Pintado‐Grima, Salvador Ventura

**Affiliations:** ^1^ Institut de Biotecnologia i de Biomedicina and Departament de Bioquímica i Biologia Molecular Universitat Autònoma de Barcelona Barcelona Spain; ^2^ Hospital Universitari Parc Taulí, Institut d'Investigació i Innovació Parc Taulí (I3PT‐CERCA) Universitat Autònoma de Barcelona Sabadell Spain

**Keywords:** Parkinson's disease, peptides, structure, therapy, α‐synuclein

## Abstract

Peptides are attracting a growing interest for therapeutic applications in biomedicine. In Parkinson's disease (PD), different human endogenous peptides have been associated with beneficial effects, including protein aggregation inhibition, reduced inflammation, or the protection of dopaminergic neurons. Such effects seem to be connected to the spatial arrangement of peptide side chains, and many of these human molecules share common conformational traits, displaying a distinctive amphipathic and cationic helical structure, which is believed to be crucial for their activities. This review delves into the relationship between these structural properties and the current evidence connecting biogenic peptides to the amelioration of PD symptoms. We discuss their implications in the disease, the different mechanisms of action, their state of validation, and their therapeutic potential.

## 1 INTRODUCTION

The global increase in life expectancy results in aging populations that are more vulnerable to suffering age‐related disorders (Hou et al. 2019). Neurodegenerative diseases such as Alzheimer's disease (AD) or Parkinson's disease (PD) are the most prevalent worldwide, with PD being the fastest‐growing neurological disorder (Dorsey et al. 2018).

PD is characterized by the loss of dopaminergic neurons in the substantia nigra pars compacta, a midbrain region related to motor control (Goetz 2011). The primary neuropathological hallmark of PD is the presence of proteinaceous intracellular deposits known as Lewis bodies and neurites, in which the main component is insoluble α‐synuclein (aSyn) (Spillantini et al. 1997; Spillantini and Goedert 2000). These protein aggregates are enriched in β‐sheet secondary structure and form highly ordered and toxic amyloid fibrils that eventually cause neuronal death (Chiti and Dobson 2017; Ke et al. 2020). There is no existing cure able to stop or delay PD progression, with current treatments providing only symptomatic relief (Pardo‐Moreno et al. 2023).

Several studies have focused on the development of organic and biological molecules able to interfere with aSyn aggregation and mitigate the neurotoxic effects of aggregated species (Kumar et al. 2021; Pandey et al. 2008; Peña‐Díaz et al. 2023; Pujols et al. 2018; Tatenhorst et al. 2016). In this context, peptide‐based strategies are garnering increasing interest due to their ability to establish more selective, specific, and stronger interactions compared to small molecules, thus reducing off‐target effects and leading to improved safety profiles (Wang et al. 2022). When compared with therapeutic proteins, like antibodies, peptides offer several advantages since their small size allows better tissue penetration and the ability to cross biological barriers more effectively; they are easier and less expensive to synthesize and manufacture, and, typically, they elicit a lower immunogenic response. Considering these benefits, different peptide‐based approaches to target aSyn‐associated pathology in PD have been explored in the last few years (Allen et al. 2023). These include peptides derived from the aSyn protein itself (especially from the N‐terminus (Horsley et al. 2022) and NAC region (El‐Agnaf et al. 2004)), as well as peptides derived from other proteins (Liang et al. 2021), library‐derived peptides (Kritzer et al. 2009), or immunogenic peptides (Mandler et al. 2014).

Many other non‐aSyn‐oriented peptides have been investigated to elucidate their neuroprotective potential in different scenarios (Dong et al. 2019), including cellular pore formation (Di Scala et al. 2022), apoptotic pathways (Shen et al. 2017), inflammation (Brown et al. 2014), or oxidation (Erfani et al. 2019). Despite the insights provided by these studies, the primary focus has been on sequence composition rather than on structural features, with only a few studies specifically dedicated to implement structure‐based peptidic therapeutic strategies, of which most are aimed at targeting aSyn (Chemerovski‐Glikman et al. 2016; Kritzer et al. 2009; Mitra and Sarkar 2020; Sangwan et al. 2020).

## 2 STRUCTURAL PEPTIDE FEATURES ARE KEY FOR ACTIVITY AND SPECIFICITY

Rationalizing structural information in peptides is fundamental for advancing their therapeutic uses in biomedicine (Iglesias et al. 2024), with applications encompassing a wide variety of activities, including antimicrobial, anticancer, antidiabetic, analgesic, immunoregulatory, or antiaggregatory peptides, among others.

aSyn architecture consists of an amphipathic N‐terminal end, a hydrophobic non‐amyloid core (NAC) responsible for amyloid formation, and an acidic C‐terminal end, which is disordered in solution. aSyn toxic species encompass oligomers and fibrils, which expose hydrophobic clusters to the solvent while retaining a highly anionic character (Fusco et al. 2017; Guerrero‐Ferreira et al. 2020). To develop a structure‐informed complementary molecule targeting aSyn toxic species selectively, we rationalized that short amphipathic and cationic peptides projecting residues' side chains in an α‐helical disposition might be effective and validated this hypothesis experimentally (Santos et al. 2021).

Recently, we demonstrated that peptides with such unique properties selectively target a specific aSyn N‐terminal motif, which is solvent exposed in the oligomer and necessary for the conversion to fibrils (Santos et al. 2024a). These peptides bind exclusively to toxic oligomers and fibrils with nanomolar affinity without recognizing the monomeric aSyn, thus preserving the protein's native function. One such peptide, LL‐37, was identified in humans, uncovering a new activity for this well‐studied antimicrobial peptide (AMP) (Santos et al. 2023).

According to rational mutagenesis studies (Santos et al. 2021; Santos et al. 2024b), the proposed peptide binding mechanism involves, first, the penetration to the dense and highly negative fuzzy coat that surrounds aSyn oligomers and fibrils thanks to their positive charge. Then, the hydrophobic face would look for and block inner aSyn hydrophobic residues, which are typically implicated in aggregation and neurotoxicity. The strong binding of this kind of peptides stems from their avidity. Once they breach the negative cloud, it is difficult that they could return back to the solution, and they are compelled to persistently engage in interactions with inner hydrophobic surfaces within oligomers and fibrils, resulting in a very low K_off_ and nanomolar K_d_ across all peptides assayed so far.

**FIGURE 1:**
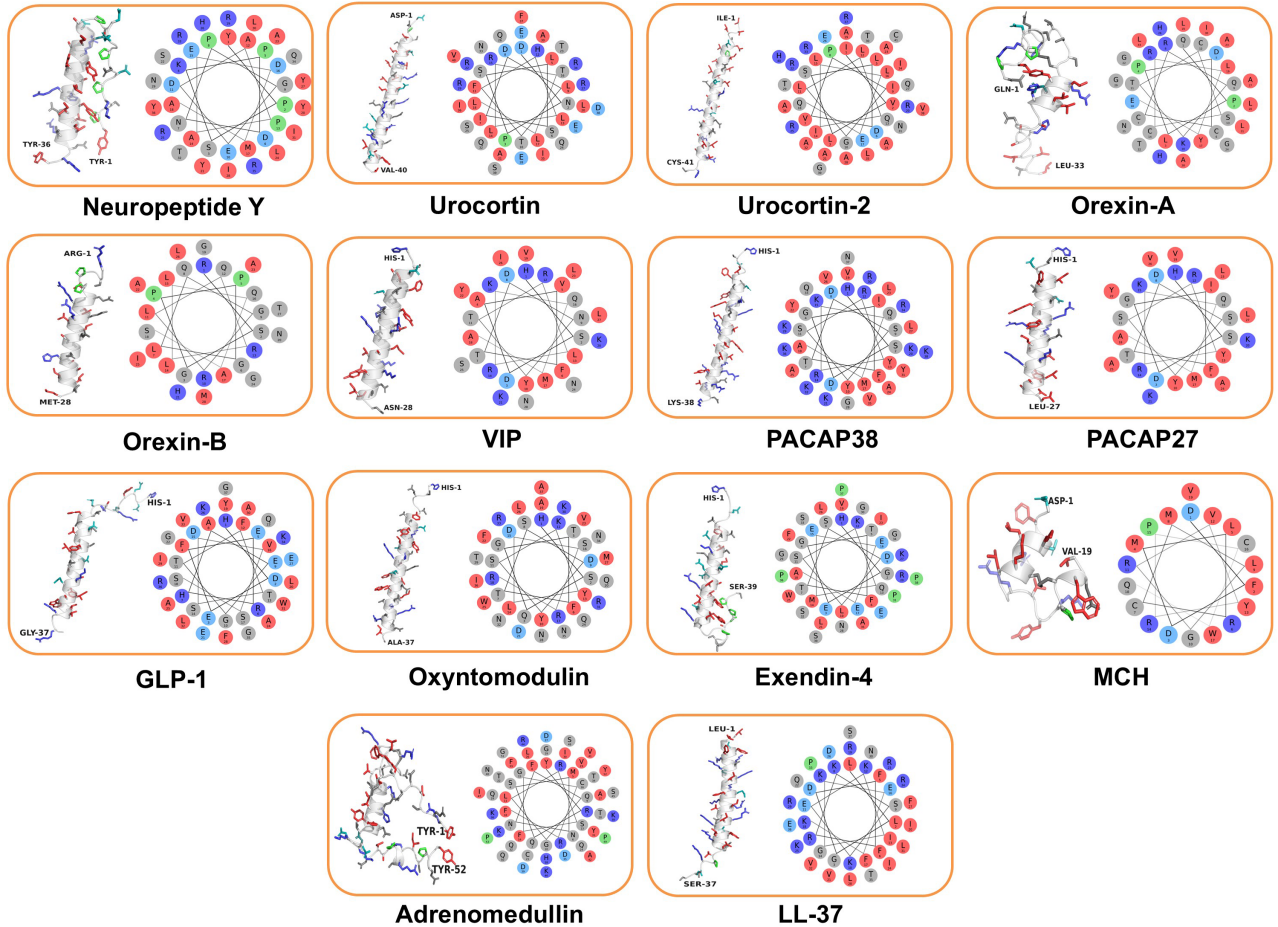
Amphipathic and cationic human helical peptides with PD associations. For each peptide, both helical wheels and predicted 3D structures (ColabFold; Mirdita et al. 2022) are represented. Red colors indicate hydrophobic amino acids whereas dark and light blues indicate cationic and anionic residues, respectively. Prolines are colored in green and the remaining neutral residues in gray.

**FIGURE 2:**
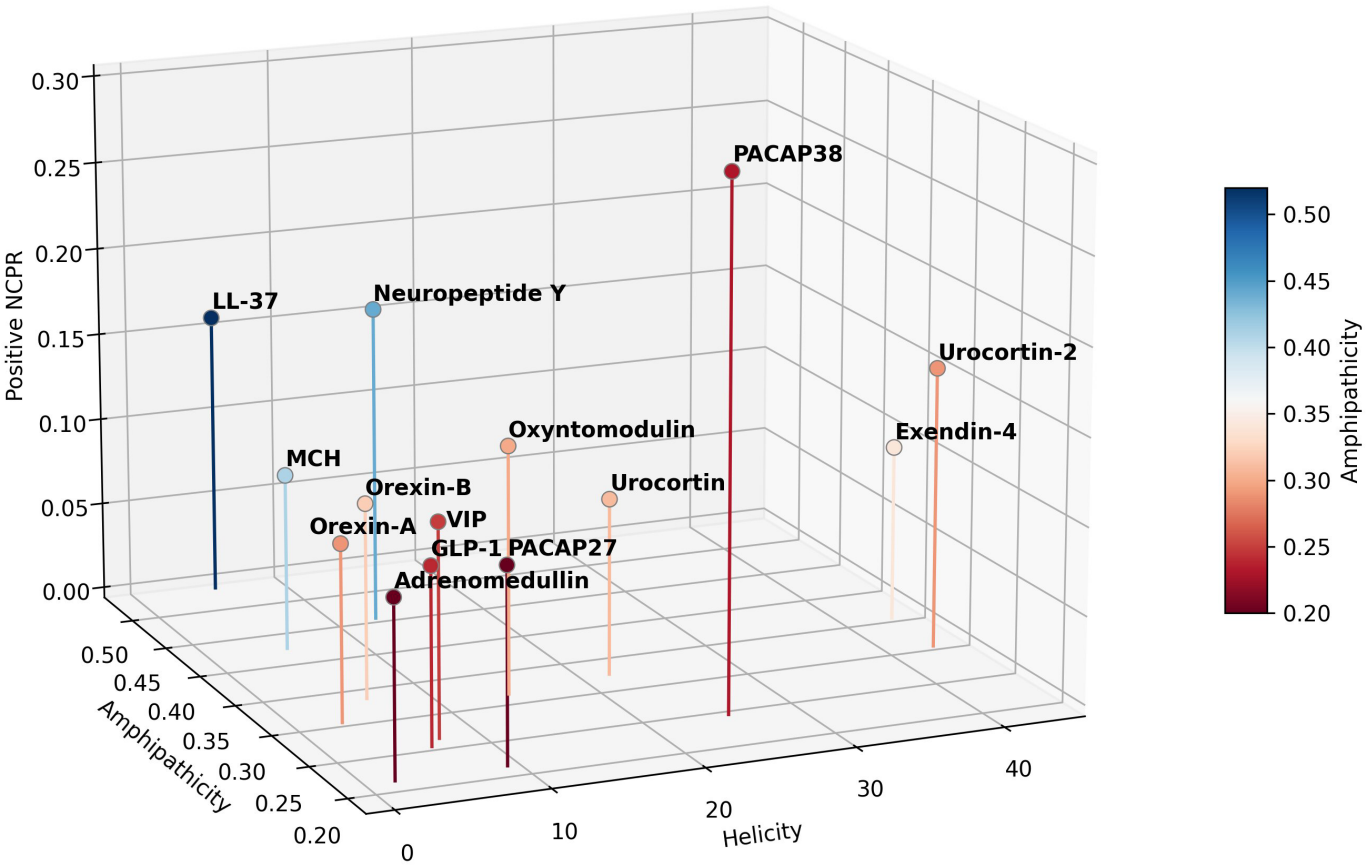
3D plot distribution of the predicted physicochemical properties for amphipathic and cationic helical peptides with previous evidences in alleviating PD symptoms.

A computational screening for additional biogenic peptides meeting the defined structural criteria in PD‐related tissues, such as the brain and gut, identified a total of 123 peptides (Pintado‐Grima et al. 2023). Notably, 14 of these, including LL‐37, had previous associations with alleviating PD symptoms, according to the literature (Figures 1 and 2). Whether these amphipathic and cationic helical peptides display aSyn inhibition activity in vivo in addition to the other described activities remains to be validated. It is plausible that peptides with such helical architecture may have different modes of action or moonlighting activities, as suggested by multiple classification predictions offered by bioinformatics tools (Bárcenas et al. 2022).

What is clear is that molecules with the described properties are somehow connected with PD and deserve further investigation. Therefore, this review aims to present the available evidence regarding the role of human amphipathic and cationic helical peptides in PD. How these peptides are implicated in disease, the contexts in which they have been validated, and their modes of action will be discussed. This novel perspective seeks to provide insights into how the structural features encoded in peptides from diverse sources can benefit PD's drug discovery in multiple ways, laying the groundwork for further therapeutic research.

## 3 AMPHIPATHIC AND CATIONIC HELICAL PEPTIDES ALLEVIATE PD SYMPTOMS IN DIFFERENT MODES

The following paragraphs describe 14 amphipathic and cationic helical peptides with previous evidence of alleviating PD symptoms. These peptides possess specific biological functions but may also have additional properties that can synergistically protect against PD. Different approaches have been used to evaluate the effect of peptides in PD. In many instances, the studies focus on both in vitro experiments and in vivo animal models of PD where neuronal PD symptoms are induced after exposure to toxic agents such as 6‐hydroxydopamine (6‐OHDA), lipopolysaccharide (LPS), or 1‐methyl‐4‐phenyl‐1,2,3,6‐tetra‐hydropyridine (MPTP) (Chia et al. 2020). The studies demonstrate different mechanisms of action (Table 1), but as the peptides share similar architectures, it is likely that their structural features would contribute to their observed protective activities.

**TABLE 1:** Neuroprotective effects observed for amphipathic and cationic human helical peptides in PD.

Peptide name	Sequence	Length	Neuroprotective effects	Mechanisms of action	Validation	References (PMID)
Neuropeptide Y	YPSKPDNPGEDAPAEDMARYYSALRHYINLITRQRY	36	Survival of dopaminergic neurons	Interaction with Y2 GPCR receptor; microglia inactivation; inhibition of ER stress	6‐OHDA mouse/rat models of PD	21816512; 30866091; 29650257
Urocortin	DNPSLSIDLTFHLLRTLLELARTQSQRERAEQNRIIFDSV	40	Prevention and recovery of nigral lesions	Autocrine/paracrine interaction with CRF‐R1/R2 receptors; inhibition microglia activation	6‐OHDA and LPS rat models of PD	17650114; 17947696
Urocortin‐2	IVLSLDVPIGLLQILLEQARARAAREQATTNARILARVGHC	41	Decrease in intracellular Ca2+; regulation of glutamatergic neurotransmission	Inhibition of voltage‐gated calcium channels and firing rate of stratium neurons.	In cellulo (rat cells); microscopy, flow cytometry and VGCC	16760921; 25837973
Orexin‐A	QPLPDCCRQKTCSCRLYELLHGAGNHAAGILTL	33	Motor deficits alleviation; sleepiness regulation	Increasing firing activity of pallidal neurons by interaction with OX1 receptors	MPTP mouse model of PD; concentration measurements in CSF	31365289; 12939433
Orexin‐B	RSGPPGLQGRLQRLLQASGNHAAGILTM	28	Motor deficits alleviation	Increasing firing activity of pallidal neurons by interaction with OX2 receptors	MPTP mouse model of PD	31365289
VIP	HSDAVFTDNYTRLRKQMAVKKYLNSILN	28	DA protection; reversion of motor symptoms	Microglia inactivation by binding VACP1 receptor; increase of GABA levels and nerve growth factors	LPS mouse model of PD; 6‐OHDA mouse model of PD	19953344; 12626429
PACAP38	HSDGIFTDSYSRYRKQMAVKKYLAAVLGKRYKQRVKNK	38	Promote DA neuron survival; improvement in behavioral deficits	Reduced microglia activation by NADPH inhibition; autophagy/apoptosis inhibition	Mesencephalic neuron–glia cultures; in vitro/MPTP mouse model of PD; 6‐OHDA rat model of PD; roteone snail model of PD	16891616; 26769362; 15084446; 18440632; 28067625
PACAP27	HSDGIFTDSYSRYRKQMAVKKYLAAVL	27	Promote DA neuron survival; improvement in behavioral deficits	Reduced microglia activation by NADPH inhibition; autophagy/apoptosis inhibition	Mesencephalic neuron–glia cultures; in vitro/MPTP mouse model of PD; 6‐OHDA rat model of PD; roteone snail model of PD	16891616; 26769362; 15084446; 18440632; 28067626
GLP‐1	HDEFERHAEGTFTSDVSSYLEGQAAKEFIAWLVKGRG	37	Arrest progression/reversion of nigral lesions	Direct interaction with GLP‐1 receptors	By homology with GLP‐1 agonists	37328112
Oxyntomodulin analogue	HSQGTFTSDYSKYLDSRRAQDFVQWLMNTKRNRNNIA	37	Prevention/reversion of motor impairments; increased dopamine synthesis	Increase tyrosine hydroxilase neurons; reduction of proinflammatory factors	MPTP mouse model, western blot, histology	26302060
Exendin‐4	HGEGTFTSDLSKQMEEEAVRLFIEWLKNGGPSSGAPPPS	39	Reduction of aSyn aggregation; improvement of motor symptoms; DA neuronal protection and restoring	Reduction of aSyn oligomers; microglial inactivation and reduced inflammation; inhibition of apoptosis; increase TH levels; inhibition of signaling pathways	Clinical trials; aSyn rat model of PD; 6‐OHDA/LPS/MPTP mouse/rat models of PD	33723752; 33433498; 19570816; 17803225
MCH	DFDMLRCMLGRVYRPCWQV	19	DA neuron protection and rescue	Activation of downstream signaling pathways by acupuncture	In vitro/in vivo 6‐OHDA and MPTP mice models; transcriptomics, quantitative PCR and western blots	27844281
Adrenomedullin	YRQSMNNFQGLRSFGCRFGTCTVQKLAHQIYQFTDKDKDNVAPRSKISPQGY	52	Neural regeneration; behavioral changes	Release of neurotrophic factors	Mouse models	18779056; 18723674
LL‐37	LLGDFFRKSKEKIGKEFKRIVQRIKDFLRNLVPRTES	37	Reduction of aSyn aggregation	Specific binding to toxic type‐B aSyn oligomers and fibrils	In vitro, ThT binding assays, dcFCC	34145261

### 3.1 Neuropeptide Y

The Neuropeptide Y (NPY) is a 36‐residue‐long peptide that has been associated with a variety of neuroprotective effects in PD and other neurodegenerative disorders such as AD and Huntington's disease (Zheng et al. 2021). In 2012, Decressac and co‐workers demonstrated that the administration of NPY increased the survival of dopaminergic (DA) neurons in both in vitro and in vivo models of PD through direct interaction with the NPY's GPCR receptor Y2 (Decressac et al. 2012). This was evidenced in a 6‐OHDA mouse model of PD treated with a Y2 antagonist and in transgenic mice lacking Y2, where the NPY had no neuroprotective effect. Activation of Y2 receptors by NPY triggered kinase signaling pathways that would promote neuronal cell survival and growth.

Another key process involving NPY is the mediation of PD‐related inflammation (Pain et al. 2019). In a rat model of PD, the administration of NPY inhibited microglial activation in the substantia nigra and reduced the number of DA neuron's deaths, suggesting a therapeutic role for NPY in preventing PD inflammation. Additionally, NPY has been associated with the inhibition of endoplasmic reticulum stress and apoptosis through downstream responses upon binding to the Y2 receptor (Lee et al. 2018), thus mitigating subsequent neuronal death. Decreased levels of NPY have been reported in the cerebrospinal fluid of PD patients (Martignoni et al. 1992). Interestingly, electroacupuncture stimulation has also proven a neuroprotective effect by increasing the levels of NPY in the substantia nigra of 6‐OHDA rat models (Yu et al. 2020).

### 3.2 Urocortin and urocortin‐2

Urocortins are a group of peptides belonging to the corticotropin‐releasing factor family, expressed in the central nervous system and peripheral tissues, that function to regulate stress responses. Urocortin is a 40‐residue long peptide that has been proposed as a potential therapeutic peptide for PD due to its ability to prevent cellular apoptosis, reduce free radicals, and mitigate neuroinflammation (Lawrence et al. 2015). In 2007, Aburmeileh and co‐workers induced PD‐like neurodegeneration in a rat model by administering 6‐OHDA and LPS (Abuirmeileh et al. 2007). The addition of urocortin significantly reduced all indicators of cellular damage, suggesting a direct neuroprotective effect. Interestingly, urocortin improved these indicators not only when provided at the same time as the toxicity inducers but also when administered 7 days later, indicating a substantial therapeutic window for the use of this peptide in recovering previously damaged DA neurons. The molecular mechanisms of action described for urocortin involve the activation of different signaling pathways upon specific interaction with its receptor in a complex network of paracrine interaction between DA neurons and microglia (Wang et al. 2007).

Following the identification of urocortin, its paralog urocortin‐2 was isolated and has also shown therapeutic potential for PD. For instance, there is evidence that urocortin‐2 decreases the levels of intracellular Ca^2+^ in rat cells via inhibition of voltage‐gated calcium channels, an activity that could regulate pathophysiological Ca^2+^ overload in PD (Tao et al. 2006). Besides, the effects of urocortin‐2 have been evaluated on striatal neurons, where the peptide was observed to inhibit spontaneous discharge and reduce the excitotoxicity of glutamatergic neurons (Liu et al. 2015a).

### 3.3 Orexin‐A and orexin‐B

Orexins are neuropeptides released in the globus pallidus, a brain region with a role in movement regulation. There are two forms of orexin: the 33‐residue long orexin‐A and a shorter 28‐residue orexin‐B that activate specific GPCR receptors, OX1R and OX2R in this region of the brain. In 2019, it was demonstrated that both orexin‐A and orexin‐B were able to alleviate motor symptoms after injection in the globus pallidus of an induced MPTP mice model of PD (Wang et al. 2019). Orexin administration increased the spontaneous firing rate of neurons from the globus pallidus, likely mediated through L‐type Ca2+ channels, suggesting it is a potential drug to treat hypokinetic motor symptoms in PD. Orexin‐A has also been associated with excessive daytime sleepiness, a common non‐motor symptom in PD. Low levels of orexin‐A have been observed in the ventricular cerebrospinal fluid of advanced PD patients (Drouot et al. 2003), and orexin has been shown to be protective against aSyn‐mediated damage to hypothalamic neurons (Bohid et al. 2024).

### 3.4 Vasoactive intestinal peptide family: VIP and PACAP

#### 3.4.1 Vasoactive intestinal peptide

The vasoactive intestinal peptide (VIP) peptide is a 28‐amino acid neuropeptide/AMP from a family that includes the pituitary adenylate cyclase‐activating polypeptide, secretin, and glucagon. VIP is secreted in the central and peripheral nervous system, and its implications in different neurological disorders, including AD and PD, have been widely studied (de Souza et al. 2021; Delgado et al. 2008; White et al. 2010). In 2003, Delgado and co‐workers demonstrated that VIP protects DA neurons in mouse embryonic neurons with LPS‐induced inflammation by inactivating microglia through binding to the VPAC1 receptor (Delgado et al. 2003). In another study, systematic VIP treatment in a 6‐OHDA rat model of PD effectively reverted the motor symptoms by preventing neuronal cell death (Korkmaz et al. 2010). These effects were associated with increased levels of the thalamic GABA neurotransmitter with an associated neuroprotection by the release of nerve growth factors. Similar outcomes were observed in a mouse model where VIP halted MPTP‐induced DA neuronal loss in the substantia nigra, preventing microglia activation in this brain region and the striatum and the release of cytotoxic mediators (Delgado and Ganea 2003).

#### 3.4.2 Pituitary adenylate cyclase‐activating polypeptide 38 and PACAP27

Pituitary adenylate cyclase‐activating polypeptide (PACAP), a neuropeptide with a high sequential identity to VIP, is widely distributed in both central and peripheral nervous systems. PACAP exists in two forms: PACAP38, which is the predominant variant in tissues, and the shorter form PACAP27, which is an N‐terminal cleaved form of PACAP38 and constitutes less than 10% of the total peptide. Numerous studies have documented the activity of PACAP as a neuronal survival factor, neuromodulator, or neuroprotectant (Arimura 1998; Arimura et al. 1994; Brenneman et al. 2002; Kozicz et al. 1997; Uchida et al. 1996).

Researchers have explored the specific association of PACAP with both motor and non‐motor symptoms of PD, focusing on different pathogenic processes. Back in 2006, it was demonstrated that PACAP38 and PACAP27 peptides exhibit neuroprotective properties by inhibiting NADPH oxidase, thereby reducing microglia‐derived oxidative stress (Yang et al. 2006). The release of proinflammatory reactive oxygen species (ROS) from microglia upon activation by environmental or endogenous factors is toxic for DA neurons. Using primary rat mesencephalic neuron–glia cultures, Yang et al. demonstrated that subpicomolar concentrations of PACAP38 and PACAP 27 protect against LPS‐induced DA neurotoxicity. The generation of ROS during neurodegeneration contributes to apoptotic and autophagic processes, causing cellular damage. Remarkably, besides the role of PACAP in microglia inactivation, it also functions as an anti‐autophagic and anti‐apoptotic peptide in both in vitro and in vivo PD models (Lamine‐Ajili et al. 2016).

The direct effects of PACAP in the degeneration of DA neurons have been studied in different animal models of the disease—including rats (Reglodi et al. 2004a; Reglodi et al. 2004b), mice (Wang et al. 2008), and snails (Maasz et al. 2017)—providing solid evidence that the peptide improves neuronal survival and alleviates behavioral symptoms. Further examination of PACAP levels in PD patients at distinct therapeutic stages of the disease revealed significant differences in the concentrations of the peptide, with lower values observed at advanced stages of the disorder (Pham et al. 2022). Overall, the growing body of evidence supporting that PACAP mediates neuroprotective effects in PD has positioned the peptide as a promising therapeutic candidate and biomarker in PD patients. However, levels of PACAP have been also associated with other cognitive diseases such as schizophrenia (Ago et al. 2018; Hashimoto et al. 2007; Vacic et al. 2011), with recent evidence correlating increased PACAP expression with schizophrenia related to suicide (Slabe et al. 2023), which indicates that therapeutic initiatives in PD should carefully monitor potential side effects.

### 3.5 Glucagon‐like peptide 1 family: GLP‐1, oxyntomodulin, and exendin‐4

#### 3.5.1 Glucagon‐like peptide 1

Glucagon‐like peptide 1 (GLP‐1) is a 30–31‐residue‐long peptide hormone that is released in the gut after food intake and promotes insulin secretion by pancreatic β‐cells. It is part of a bigger family of glucagon‐derived peptides that share similar endocrine activities. Given their ability to regulate insulin secretion, GLP‐1, and GLP‐1 analogs were first studied as antidiabetic drugs for type II diabetes (Maselli and Camilleri 2021). The increasing body of evidence associating metabolic disorders and neurodegenerative diseases opened a new avenue for repurposing peptides from the GLP‐1 family in PD (Nowell et al. 2023). A recent study in a transgenic mouse model of PD suggests that enhancing GLP‐1 secretion in the enteric nervous system offers central and enteric neuroprotection against synucleinopathy‐induced neurodegeneration (Pradeloux et al. 2024). Actually, stimulation of GLP‐1 receptors has been exploited to reverse key deficits in distinct rodent models of PD (Harkavyi et al. 2008; Nowell et al. 2023). However, given the variability of endogenous peptides that can bind to GLP‐1 receptors, many studies and clinical assays have focused on identifying receptor agonists or GLP‐1 analogs that could bind with higher affinities and specificity (Kopp et al. 2022). At least six GLP‐1 receptor agonists have been or are being tested as potential treatments in persons with PD (McFarthing et al. 2022); among them is the peptide lixisenatide, which has demonstrated efficacy in attenuating motor impairment and preventing the loss of dopamine neurons in an MPTP mouse model of PD (Liu et al. 2015b). A phase II clinical trial in persons with early PD showed a reduction in the progression of motor disability, although gastrointestinal side effects were reported (Meissner et al. 2024).

#### 3.5.2 Oxyntomodulin

Oxyntomodulin is a 37‐amino acid hormone that is produced by cleavage of the preproglucagon peptide. Similar to GLP‐1, it contains a glucagon sequence with an extension that helps it to bind GLP‐1 receptors with low affinity, while exhibiting a longer half‐time than GLP‐1 in the bloodstream. Given the modularity of this family of peptides, oxyntomodulin analogues have been synthesized to enhance the protease resistance, extending their activity in the blood to hours in vivo. The neuroprotective effects of one of such analogues, D‐Ser2‐oxyntomodulin (Oxy), have been evaluated in an MPTP‐mouse model of PD (Liu et al. 2015c). Liu and co‐workers demonstrated that Oxy normalized or reduced different reporters of motor impairment, including locomotor activity, sensory‐motor control, swimming activity, and muscle strength. Oxy also normalized the levels of the tyrosine hydroxylase enzyme in the substantia nigra and the striatum, responsible for dopamine synthesis, as well as levels of pro‐inflammatory factors.

These findings support further therapeutic exploration of both Oxy and Oxyntomodulin. Indeed, Oxy ameliorated Aβ31‐35‐induced circadian rhythm disorder in an AD mouse model (Wang et al. 2020). The original Oxyntomodulin has also been shown to have neurotrophic and neuroprotective effects in neuronal cells and a rat model of stroke (Li et al. 2017).

#### 3.5.3 Exendin‐4

Exendin‐4 is a GLP‐1 analog peptide found in the saliva of venomous Gila monster (*Heloderma suspectum*). It is a 39‐residue peptide whose unique amino acid composition prevents enzyme cleavage and extends peptide's half‐life in plasma. In light of these properties and its GLP‐1 activity in regulating insulin secretion, in 2005, exendin‐4 (marketed as exenatide) became the first peptide approved by the FDA for the treatment of type II diabetes (Davidson et al. 2005). Given the claimed neuroprotection of different GLP‐1 agonists, there exists a high interest in the therapeutic potential of this peptide for neurodegenerative diseases such as PD or AD (Verma et al. 2024). A wealth of studies has explored the neuroprotective effects of exendin‐4 in different animal models of PD, with findings that include microglial deactivation, suppression of inflammation, improvement of motor symptoms, stimulation of neurogenesis, restoration of dopamine levels or prevention of DA neuronal loss (Bertilsson et al. 2008; Kim et al. 2009; Li et al. 2009; Verma et al. 2024). Interestingly, some of these studies were conducted in aSyn‐mediated models of PD (Bergkvist et al. 2021; Bu et al. 2021), where exendin‐4 treatment was found to reduce levels of aggregated aSyn by inhibiting cellular signaling pathways. In particular, in 2021, Zhang and co‐workers reported reduced levels of aSyn oligomers after exendin‐4 treatment in a 6‐OHDA model of PD (Zhang et al. 2021). Additionally, in a mouse model of PD, lipid nanoparticles functionalized with exenatide improved motor symptoms and increased dopamine levels in substantia nigra pars compacta and the striatum, with a concomitant reduction of as aSyn deposition into Lewy bodies (Wu et al. 2024).

The therapeutic potential of exendin‐4 is evident from the numerous clinical trials currently being conducted on it for various neurodegenerative diseases. In an uncompleted AD trial, reduced levels of Aβ‐42 in plasma neuronal extracellular vesicles were observed in patients who received exenatide, suggesting it decreased brain amyloidosis (Mullins et al. 2019). In PD, a pilot clinical trial revealed improvement of motor and cognitive symptoms lasting up to 12 months post‐treatment with the peptide (Aviles‐Olmos et al. 2014). This positive outcome enabled the researchers to secure funding for a Phase II study (NCT01971242). Again, the improvements persisted even after the drug was cleared from the body, indicating that the peptide had disease‐modifying effects in this cohort of PD patients.

### 3.6 Melanin‐concentrating hormone

As for NPY, acupuncture treatment has been reported to regulate the levels of melanin‐concentrating hormone (MCH) peptides and relieve PD symptoms (Park et al. 2017). Park et al. demonstrated that hypothalamic MCH was overexpressed after acupuncture treatment in a mouse model of PD and that this effect projected to the substantia nigra. They observed a functional protective role for MCH in primary neuronal cultures and discovered DA neuron rescue following peptide administration in 6‐OHDA or MPTP models of PD. Interestingly, PD patients present decreased levels of hypothalamic neurons expressing MCH peptides, causing neuroendocrine dysregulation, impacting circadian function (Willis 2008), and potentially contributing to non‐motor features in this disease.

### 3.7 Adrenomedullin

Adrenomedullin (AM) is a 52‐residue vasodilator neuropeptide hormone that has been shown to exert neuroprotective effects in various models of neurological damage (Li et al. 2020). AM contributes to reducing oxidative stress, inflammation, and apoptosis in neurons playing a crucial role in brain injury and neural regeneration processes. It can influence different neurotransmitter systems, including those involving dopamine and glutamate, which are dysregulated in PD. Brain levels of AM have been correlated with behavioral changes in mouse models, and mice lacking AM showed impaired motor coordination and anxiety under stress conditions (Fernández et al. 2008).

The carotid body (CB) is an alternative dopaminergic tissue. Autotransplantation of CB into the striatum of rodent and nonhuman primate models of PD has shown trophic protection and restoration of the dopaminergic nigrostriatal pathway (Villadiego et al. 2023). It has been proposed that the CB secretes a growth factor that induces recovery of the original neural pattern, restoring the levels of dopamine. AM is considered a strong candidate for this function due to its abundant presence in the CB and its characteristics as a potent growth factor (Martínez et al. 2003). Additionally, other neuropeptide modulators sharing the here‐discussed fold, such as NPY, PACAP, and VIP, are also present in the CB and can contribute to the neuroregenerative action of CB grafts (Porzionato et al. 2008).

## 4 DISCUSSION AND OUTLOOK

**FIGURE 3:**
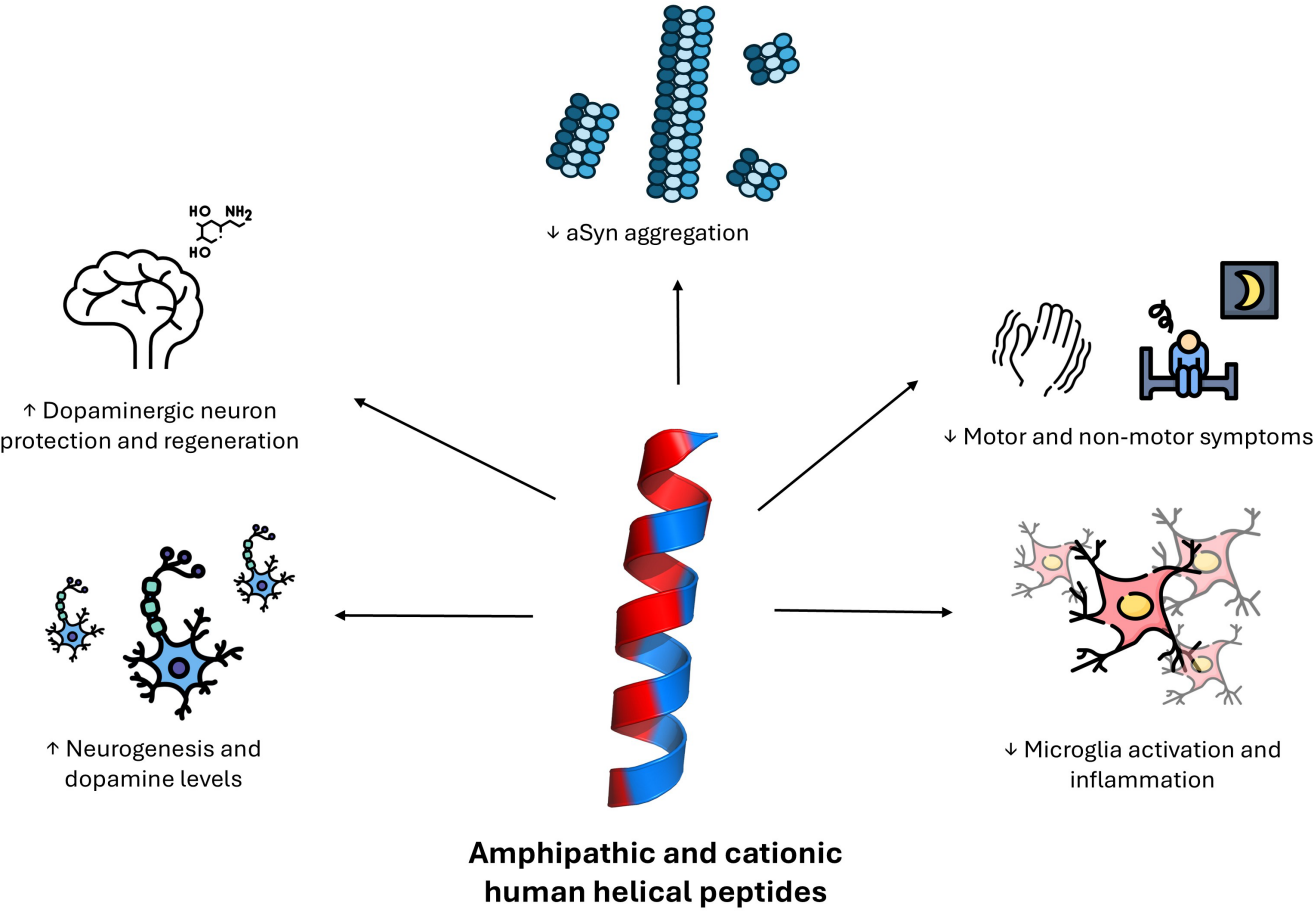
Schematic representation of the main associated neuroprotective effects observed for amphipathic and cationic helical peptides in PD. Figure created with resources of Flaticon.com.

Peptides are gaining significant interest for therapeutic applications due to their natural amino acid composition, bioavailability, specificity, low costs, and reduced side effects (Wang et al. 2022). Traditionally, most studies have focused on peptide sequences, often overlooking the crucial impact of their conformation on biological activity (Iglesias et al. 2024). In this review, we have covered the described roles of selected amphipathic and cationic helical peptides in PD, a fold that seems to be associated with neuroprotection in front of aSyn toxic species (Santos et al. 2021). These peptides have demonstrated diverse beneficial effects, such as protecting dopaminergic neurons, reducing inflammation, and mitigating autophagy, which collectively contributes to improvements in both motor and non‐motor symptoms of PD, as summarized in Figure 3. However, most studies have investigated the peptides' activity in conditions mimicking the late phases of the disease rather than focusing on their role in disease onset and progression. This is important because the shared structural fold of biogenic peptides of this kind has the potential to bind and inhibit aSyn toxic oligomers and fibrils, thus influencing one of the underlying causes of PD (Pintado‐Grima et al. 2023). Despite only LL‐37 (Santos et al. 2021) and exendin‐4 (Bu et al. 2021) have been experimentally validated to inhibit aSyn aggregation in vitro, preliminary data from our lab support an anti‐Syn activity for all the peptides in this structural class we have analyzed so far. Still, in the present review we have specifically focused on the neuroprotective effects of biogenic amphipathic and cationic helical peptides already documented in the literature. These effects are mediated by diverse molecular pathways, often involving interactions with different families of neuronal receptors. Note that, whether peptides with the described structural properties are likely to interact with aSyn toxic species, they should not generically bind to the same receptors as specific sequential features are required for these contacts to avoid mis‐signaling.

Overall, it seems that the discussed peptides could have multiple neuroprotective functions that are displayed on top of a common 3D architecture and work synergistically to alleviate PD symptoms in different ways, making them of significant therapeutic interest. Beyond the proposed anti‐aSyn activity, amphipathic and cationic helical peptides are prone to interact with cellular membranes. Indeed, all described peptides have membrane binding segments predicted by dedicated protein–membrane interaction tools (Table 2 and Figure S1, Supporting Information). Since aSyn toxic oligomers can perturb and disrupt biological membranes (Fusco et al. 2017), peptides could exert their effect not only by binding to oligomers and fibrils but also by competing in membrane binding while avoiding subsequent oligomer‐derived neurotoxicity. Indeed, an initiative to target aSyn aggregation in the gut by administering a small molecule (ENT‐01) aimed to displace αSyn from cell membranes (Hauser et al. 2019) has recently demonstrated neuroprotective activity in a randomized clinical trial of PD (Camilleri et al. 2022).

**TABLE 2:** Protein–membrane interaction predictions by PMIPred (van Hilten et al. 2024).

Peptide name	Avg ΔΔF_adj	Lowest ΔΔF_adj	N sensing	N binding	N non‐binding	Sequence lowest ΔΔF_adj
Neuropeptide Y	−6.45	−17.32	4	8	10	19‐RYYSALRHYINLITR‐33
Urocortin	−8.3	−19.08	3	11	11	9‐LTFHLLRTLLELART‐23
Urocortin‐2	−8.91	−19.41	3	11	13	2‐VLSLDVPIGLLQILL‐16
Orexin‐A	−9.34	−14.8	9	7	3	17‐YELLHGAGNHAAGIL‐31
Orexin‐B	−11.1	−15.92	7	7	0	1‐RSGPPGLQGRLQRLL‐15
PACAP38	−10.73	−16.7	6	16	2	17‐MAVKKYLAAVLGKRY‐31
PACAP27	−9.39	−13.0	5	6	2	13‐YRKQMAVKKYLAAVL‐27
VIP	−9.0	−12.7	8	4	2	13‐LRKQMAVKKYLNSIL‐27
MCH	−16.92	−18.28	0	5	0	5‐LRCMLGRVYRPCWQV‐19
Oxyntomodulin analogue	−7.81	−12.8	7	7	9	12‐KYLDSRRAQDFVQWL‐26
GLP‐1	−5.74	−15.24	3	6	14	18‐SYLEGQAAKEFIAWL‐32
Adrenomedullin	−8.54	−15.73	3	19	16	8‐FQGLRSFGCRFGTCT‐22
Exendin‐4	−7.73	−14.87	5	10	10	12‐KQMEEEAVRLFIEWL‐26
LL‐37	−12.48	−21.68	7	15	1	18‐KRIVQRIKDFLRNLV‐32

*Note*: PMIPred provides numerical output on the curvature‐sensing free energy (ΔΔF_adj) for each consecutive 15‐residue window along the sequence and the assessment of their binding (interaction with curved and flat membranes), sensing (interaction with curved membranes), or non‐binding (no interaction with membranes) propensities. ΔΔF_adj > −6.4 for non‐binders, −10.0 ≤ ΔΔF_adj ≤ −6.4 for sensors and ΔΔF_adj < −10.0 for binders. The number of sensing, binding, and non‐sensing segments is specified for each peptide as well as the sequence of the best (lowest ΔΔF_adj) binding segment.

The therapeutic potential for this family of biogenic peptides is evidenced by numerous ongoing clinical trials and registered patents in which they are involved. Some of them have already received FDA approval for other disorders and thus are suitable candidates for repurposing in the treatment of brain diseases.

Although promising preliminary data suggest this direction, experimental validation is still required to prove the generic novel activity of amphipathic and cationic helical peptides (anti‐aggregation by binding to toxic aSyn species), discussed in this review. Specifically, it is essential to correlate the in vivo reported neuroprotective effects with a reduction of aSyn fibrils and oligomer binding in critical brain regions. Given the peptides' potential to rapidly interact with the early toxic species (type‐B oligomers) in the aSyn aggregation cascade, their activity should be preferentially tested in new animal models specifically designed to study the earliest stages of PD, with a focus on aSyn pathology (Muñoz‐Juan et al. 2024; Richter et al. 2023). If effective in these models, early administration of the peptides before the onset of motor symptoms could potentially slow disease progression. Should these studies succeed, amphipathic and cationic helical peptides could be leveraged as both therapeutic agents and biomarkers. The levels of some of such peptides appear to relate to the disease stage, suggesting they may act as natural guardians, maintaining homeostasis in the brain, potentially delaying PD‐associated neurodegeneration.

## AUTHOR CONTRIBUTIONS

**Carlos Pintado‐Grima:** Conceptualization; investigation; writing – original draft; writing – review and editing; methodology; formal analysis. **Salvador Ventura:** Conceptualization; investigation; funding acquisition; writing – review and editing; project administration; supervision; formal analysis.

## Supporting information


**Data S1.** Supporting Information.
