# Supplementary material for: The role of amphipathic and cationic helical peptides in Parkinson's disease
Source: Protein Sci. 2024 Dec 25;34(1):e70020. doi: 10.1002/pro.70020 (PMC11669119; doi:10.1002/pro.70020)
Supplement: Supplementary file 1 — Data S1. Supporting Information. [file PRO-34-e70020-s001.pdf]

Supplementary material for *The Role of Amphipathic and Cationic Helical Peptides in Parkinson's Disease*

Carlos Pintado-Grima and Salvador Ventura

Supplementary S1: membrane binding predictions provided by PMIPred (https://pmipred.fkt.physik.tu-dortmund.de/) with default parameters for full-length biogenic amphipathic and cationic helical peptides. We selected PIMIPred because: i) it focuses on the prediction of interacting segments rather than single amino acids, ii) it was trained with peptides, iii) it allows for PDB input, iv) provides free energy quantitative output and v) it is sensitive to the membrane curvature. PMIPred provides both graphical and numerical output on the predicted sensing and binding regions. Numerical output include free energy calculations for each consecutive 15-residue window along the sequence and the assessment of their binding, sensing or non-binding propensities. Red, orange and purple indicate binding, sensing and non-binding/below SASA threshold residues, respectively.

Neuropeptide Y

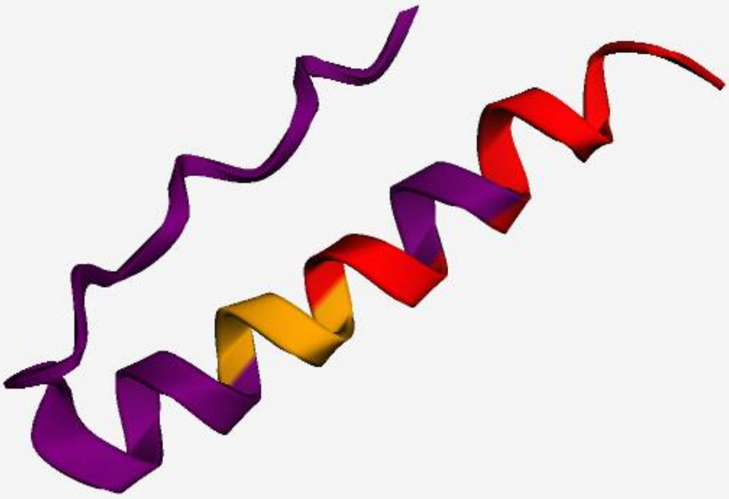

**Chain**	AAAAAAAAAAAAAAAAAAAAAAAAAAAAAAAAAAAA
**#n**	1........10........20........30.....
**Sequence**	YPSKPDNPGEDAPAEDMARYYSALRHYINLITRQRY
**Classification (if accessible)**	------------------SSSSBBB---BBBBBBBB

Urocortin

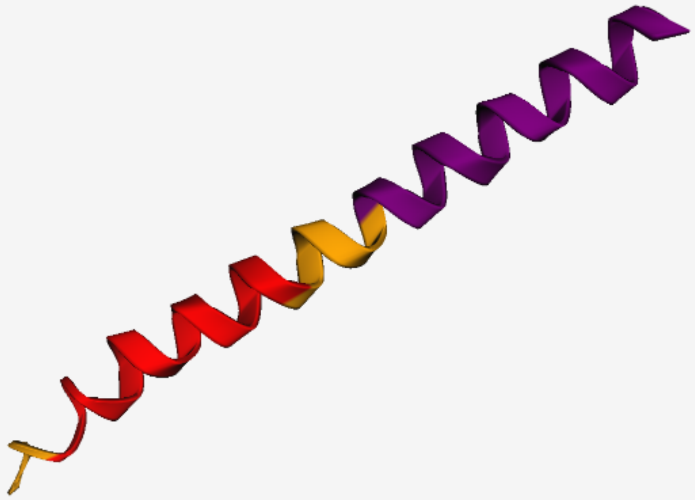

**Chain**	AAAAAAAAAAAAAAAAAAAAAAAAAAAAAAAAAAAAAAAA
**#n**	1........10........20........30........40
**Sequence**	DNPSLSIDLTFHLLRTLLELARTQSQRERAEQNRIIFDSV
**Classification (if accessible)**	SSSBBBBBBBBBBBBBBBSSSSS-----------------

Urocortin-2

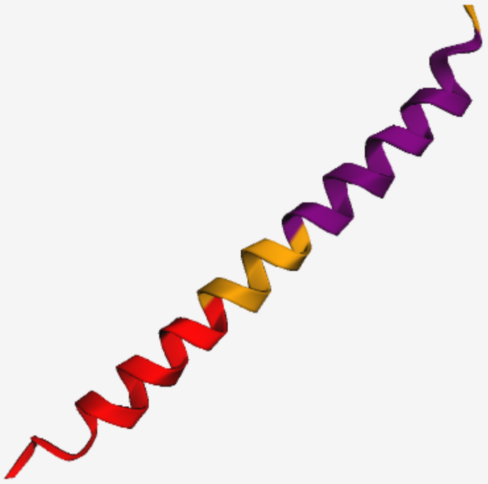

**Chain**	AAAAAAAAAAAAAAAAAAAAAAAAAAAAAAAAAAAAAAAAA
**#n**	1........10........20........30........40
**Sequence**	IVLSLDVPIGLLQILLEQARARAAREQATTNARILARVGHC
**Classification (if accessible)**	BBBBBBBBBBBBBBBBSSSSSSS-----------------S

Orexin-A

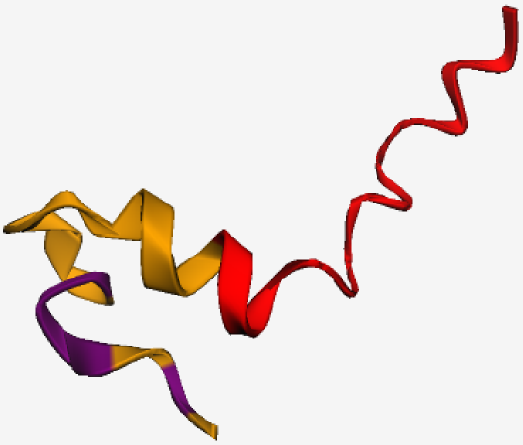

**Chain**	AAAAAAAAAAAAAAAAAAAAAAAAAAAAAAAAA
**#n**	1........10........20........30..
**Sequence**	QPLPDCCRQKTCSCRLYELLHGAGNHAAGILTL
**Classification (if accessible)**	S-S----SSSSSSSSSSSBBBBBBBBBBBBBBB

Orexin-B

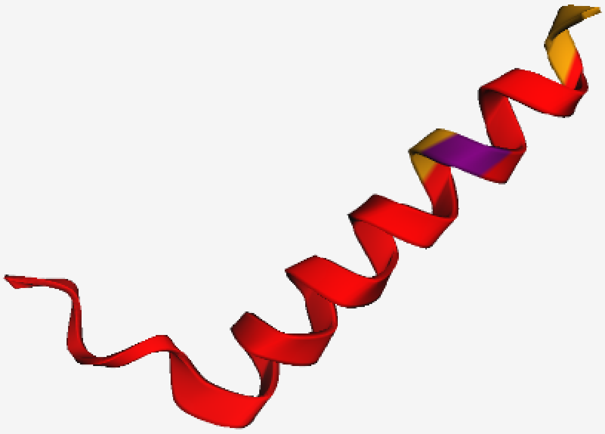

**Chain**	AAAAAAAAAAAAAAAAAAAAAAAAAAAAA
**#n**	1........10........20.......
**Sequence**	RSGPPGLQGRLQRLLQASGNHAAGILTM
**Classification (if accessible)**	BBBBBBBBBBBBBBBBBBBS-BBBBBSS

PACAP38

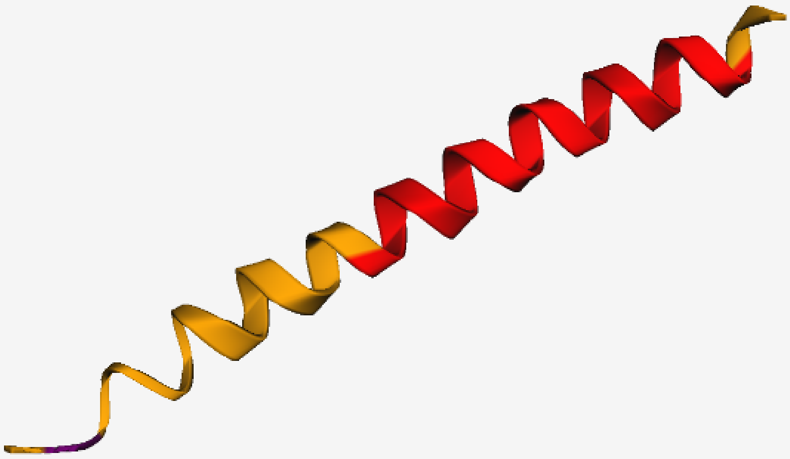

**Chain**	AAAAAAAAAAAAAAAAAAAAAAAAAAAAAAAAAAAAAA
**#n**	1........10........20........30.......
**Sequence**	HSDGIFTDSYSRYRKQMAVKKYLAAVLGKRYKQRVKNK
**Classification (if accessible)**	S-SSSSSSSSSSSSSSBBBBBBBBBBBBBBBBBBBBSS

PACAP27

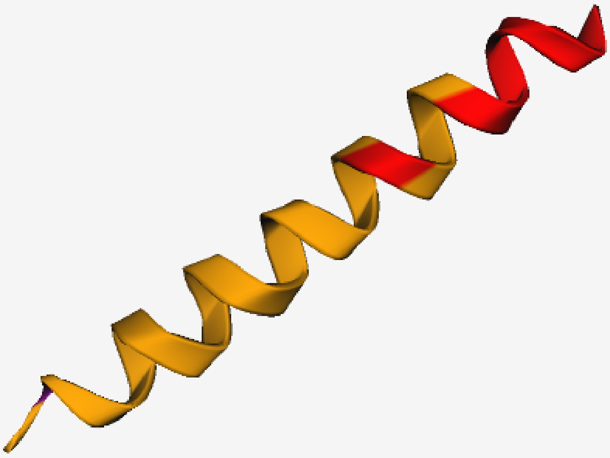

**Chain**	AAAAAAAAAAAAAAAAAAAAAAAAAAA
**#n**	1........10........20......
**Sequence**	HSDGIFTDSYSRYRKQMAVKKYLAAVL
**Classification (if accessible)**	S-SSSSSSSSSSSSSSSBSSSBBBBBB

VIP

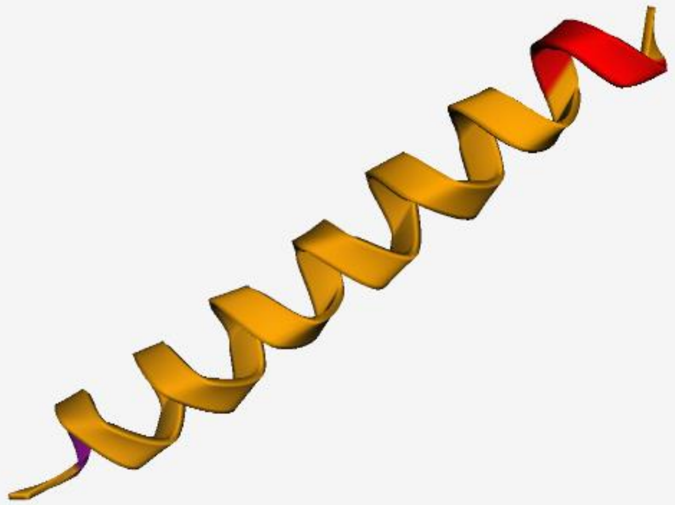

Chain	AAAAAAAAAAAAAAAAAAAAAAAAAAAA
#n	1........10........20.......
Sequence	HSDAVFTDNYTRLRKQMAVKKYLNSILN
Classification (if accessible)	S-SSSSSSSSSSSSSSSSSSSSSSBBBS

MCH

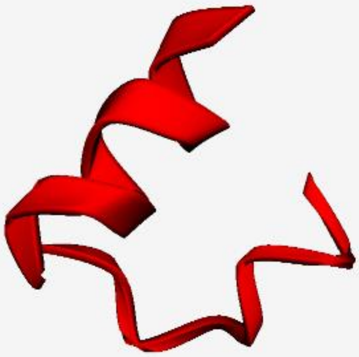

Chain	AAAAAAAAAAAAAAAAAAA
#n	1........10........
Sequence	DFDMLRCMLGRVYRPCWQV
Classification (if accessible)	BBBBBBBBBBBBBBBBBBB

Oxyntomodulin analogue

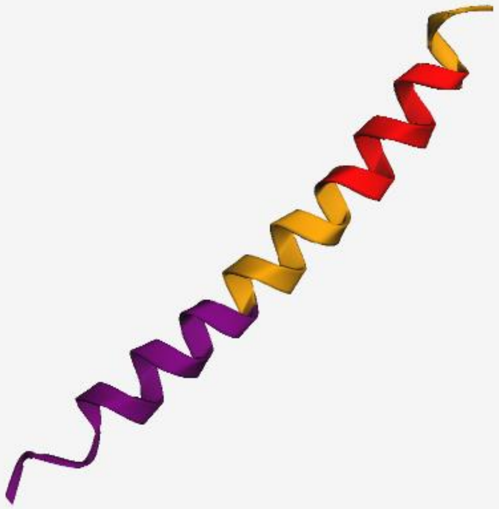

**Chain**	AAAAAAAAAAAAAAAAAAAAAAAAAAAAAAAAAAAAA
**#n**	1........10........20........30......
**Sequence**	HSQGTFTSDYSKYLDSRRAQDFVQWLMNTKRNRNNIA
**Classification (if accessible)**	---------------SSSSSSSSSBBBBBBBBBSSSS

GLP-1

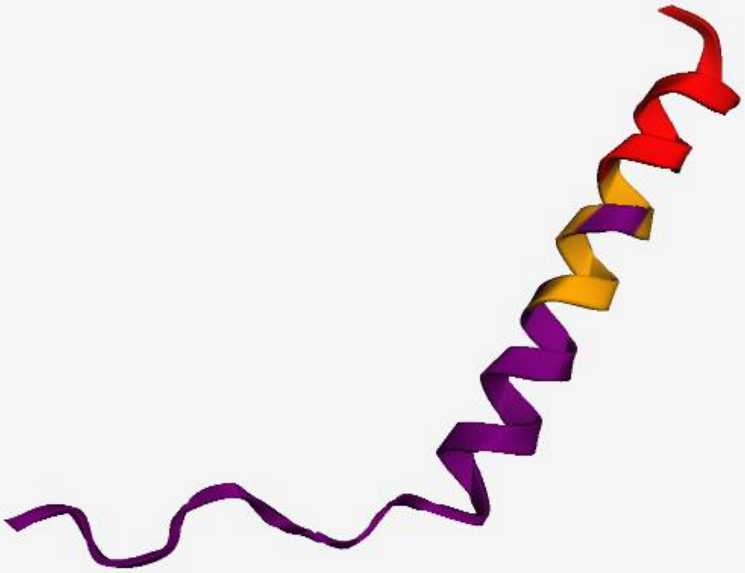

**Chain**	AAAAAAAAAAAAAAAAAAAAAAAAAAAAAAAAAAAAA
**#n**	1........10........20........30......
**Sequence**	HDEFERHAEGTFTSDVSSYLEGQAAKEFIAWLVKGRG
**Classification (if accessible)**	---------------------SSSS-SSBBBBBBBBB

Adrenomedullin

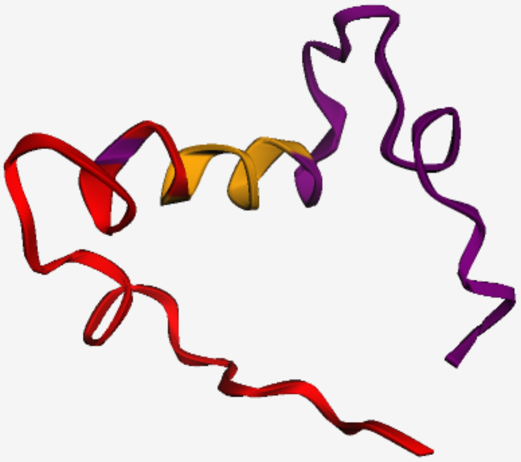

Chain	AAAAAAAAAAAAAAAAAAAAAAAAAAAAAAAAAAAAAAAAAAAAAAAAAAAA
#n	1........10........20........30........40........50.
Sequence	YRQSMNNFQGLRSFGCRFGTCTVQKLAHQIYQFTDKDKDNVAPRSKISPQGY
Classification (if accessible)	BBBBBBBBBBBBBBBBBBBBBB-BBSSSSSS---------------------

Exendin-4

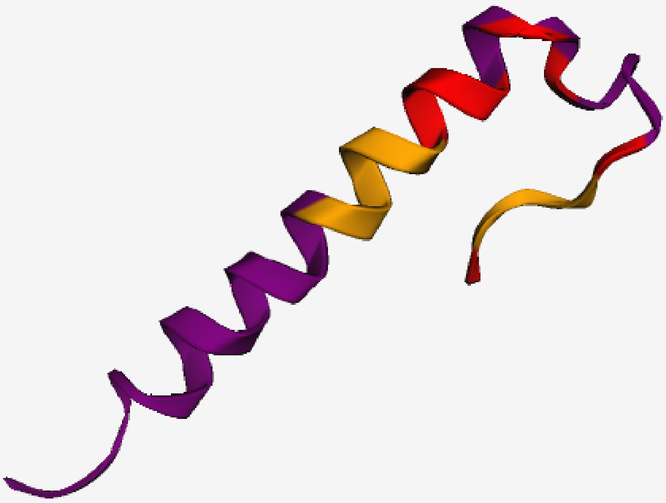

Chain	AAAAAAAAAAAAAAAAAAAAAAAAAAAAAAAAAAAAAAA
#n	1........10........20........30........
Sequence	HGEGTFTSDLSKQMEEEAVRLFIEWLKNGGPSSGAPPPS
Classification (if accessible)	----------------SSSSSBBBB--B-B----BSSSB

LL-37

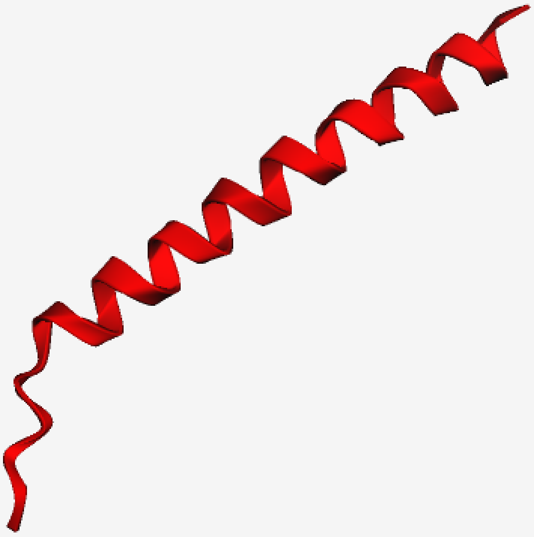

**Chain**	AAAAAAAAAAAAAAAAAAAAAAAAAAAAAAAAAAAAA
**#n**	1........10........20........30......
**Sequence**	LLGDFFRKSKEKIGKEFKRIVQRIKDFLRNLVPRTES
**Classification (if accessible)**	BBBBBBBBBBBBBBBBBBBBBBBBBBBBBBBBBBBBB
